# Exploring multilevel barriers and facilitators to antiretroviral therapy adherence among adults living with HIV in ART clinic in Mogadishu, Somalia: a qualitative study guided by the socio-ecological model

**DOI:** 10.1186/s12981-025-00830-9

**Published:** 2025-11-29

**Authors:** Mohamed Jayte, Abdifitah Abdullahi Mohamed, Abdifatah Karshe, Farah Dubad Abdi, Fathi Ali Araye, Ahmed Shafie Adan

**Affiliations:** 1 Department of Internal Medicine, Kampala International University, P.O. Box 7062, Kampala, Uganda; 2 Department of Microbiology, Kampala International University, Kampala, Uganda; 3 Department of Psychiatric and Mental Health, Kampala International University, Kampala, Uganda; 4 Department of Cardiology at Somali Türkiye Training and Research, Mogadishu, Somalia

**Keywords:** Antiretroviral therapy, HIV, Adherence, Barriers, Facilitators, Stigma, Somalia, Qualitative study

## Abstract

**Background:**

Antiretroviral therapy (ART) has dramatically reduced HIV-related morbidity and mortality worldwide, but adherence remains a critical challenge, particularly in Sub-Saharan Africa. In Somalia, where HIV prevalence is low but health systems are fragile, little is known about the lived experiences of people living with HIV (PLHIV) regarding ART adherence.

**Methods:**

A qualitative study was conducted at ART clinic In Mogadishu Somalia between March–August 2024. A total of 22 adult PLHIV and 12 healthcare providers (8 ART nurses and 4 case managers) participated. Purposive sampling was used to recruit participants; Data were collected through 30 in-depth interviews and 4 focus group discussions using semi-structured guides. Interviews were audio-recorded, transcribed, translated, and thematically analyzed using NVivo software, with emerging themes classified into barriers and facilitators of adherence.

**Results:**

Barriers to adherence included stigma and discrimination, religious and cultural beliefs (such as reliance on prayer or holy water), economic hardship (transport costs, food insecurity), health system challenges (stock-outs, long waiting times, confidentiality concerns), and psychological factors (depression, denial). Facilitators included strong family and social support, religious coping that motivated adherence, positive patient–provider relationships, peer support networks, and personal motivation and resilience, particularly among patients committed to living for their children. Illustrative quotes highlighted the daily struggles and strategies patients employed to remain in care.

**Conclusion:**

ART adherence in Somalia is shaped by complex interactions between socio-cultural, economic, psychological, and health system factors. Interventions that reduce stigma, integrate mental health services, strengthen health systems, provide economic and food support, and leverage religious and peer networks are essential. Collaborating with traditional and religious leaders may further improve acceptance and sustainability of ART programs.

## Background

Antiretroviral therapy (ART) has substantially reduced HIV-related morbidity and mortality globally by improving immune function and preventing opportunistic infections [1]. However, the success of ART programs hinges on high levels of adherence and continuous retention in care—typically requiring ≥ 95% of doses taken as prescribed [2]. Poor adherence undermines viral suppression, fosters drug resistance, and increases transmission risk, threatening public health investments [3].

Despite advancements in HIV management, non-adherence remains a pervasive challenge worldwide. A systematic review identified key barriers such as stigma, economic hardship, food insecurity, mental health disorders, and limited healthcare access; concurrently, supportive relationships, culturally informed interventions, and empowerment of people living with HIV (PLHIV) emerged as effective facilitators [4]. Global strategies therefore emphasize multidisciplinary, patient-centered interventions that address these social and structural determinants [5].

Sub-Saharan Africa—which bears nearly two-thirds of the global HIV burden—continues to experience adherence barriers amplified by poverty, transport constraints, stigma, and weak health systems [6]. For example, in rural Mozambique, patients cited food insecurity, transport difficulties, and lack of confidentiality as major obstacles to consistent ART adherence [7]. Similarly, a scoping review across SSA highlighted the importance of integrating mental health, flexible clinic hours, peer support, and mobile health tools to improve adherence [8].

Although Somalia has one of the lowest HIV prevalence rates in Africa (estimates ranging from 0.3% to 0.7% among adults) [9, 10], the fragile health system, decades of conflict, and limited infrastructure pose unique challenges to HIV care. Estimates suggest approximately 8,200 people are living with HIV in Somalia, with very low ART coverage and persistently limited awareness, According to UNAIDS [11], only about 42% of PLHIV in Somalia were receiving ART, and viral suppression data remain unavailable due to limited monitoring systems. National program reports estimate adherence below 70% and retention at 12 months under 60%, underscoring urgent need for contextual understanding [12, 13]. A recent hospital-based study in Mogadishu reported 30.5% non-adherence among PLHIV, with contributing factors including stigma, transport issues, side effects, and social isolation [13]. Misconceptions about HIV transmission are also widespread; one survey found over two-thirds of Somali women held inaccurate beliefs, driven by low education and limited media exposure [14].

While quantitative evidence highlights notable non-adherence rates and associated factors, there is a critical gap in understanding the lived experiences, beliefs, and contextual influences surrounding ART adherence among Somali adults. There is limited qualitative insight into how stigma, faith, economic hardship, coping strategies, and health system factors shape adherence behaviors in Somalia’s distinct socio-cultural and conflict-affected environment.

Quantitative studies have provided numerical estimates, but they overlook how social, cultural, and systemic realities interact to shape adherence behaviors. This qualitative study fills that gap by deeply exploring both barriers and facilitators to ART adherence among HIV-positive adults in Somalia. Understanding personal narratives and provider perspectives is essential to inform culturally appropriate, context-specific interventions—such as engaging religious leaders, community-based outreach, peer support, and structural supports—that are feasible and acceptable within Somali society. Tailoring ART adherence strategies to local realities is vital to improving outcomes for PLHIV and strengthening Somalia’s HIV response.

## Methods

### Study setting

This study was carried out at Banadir Hospital, Mogadishu. The hospital serves a catchment population of approximately half million people. The hospital began providing ART services in 2006, following the national scale-up of free ART programs supported by the Somali Ministry of Health and international partners. At the time of data collection, over 200 patients were receiving ART at Banadir Hospital.

According to national guidelines, adult patients with HIV infection and CD4 count ≤ 350 cells/mm³, or with any clinical symptoms indicating WHO stage 3 or 4 disease irrespective of CD4 count, are eligible to start ART. At both hospitals, dedicated ART nurses provide counseling before and after ART initiation, while physicians initiate ART based on clinical and laboratory findings. Nurses review patients monthly during the first six months of ART and every three months thereafter. Patients with opportunistic infections (OIs) or adverse drug reactions are referred to physicians.

The study sites also have peer-support programs staffed by case managers who are themselves living with HIV and receiving ART. ART nurses refer patients to case managers for adherence counseling and management of ART-related risk factors. Case managers are responsible for tracing patients lost to follow-up through community visits and telephone contact, in addition to providing ongoing psychosocial support.

### Inclusion and exclusion criteria

- *Patients*: Adults (≥ 18 years) who had been receiving ART for at least one month and could provide informed consent in Somali were eligible for interviews.
- *Providers*: All ART nurses and case managers who had worked in ART clinics for at least six months were invited to participate in FGDs.

Patients who were severely ill, bedridden, or unable to communicate were excluded.

### Recruitment and sampling

A purposive sampling approach was used to ensure diversity in adherence history, gender, and duration on ART, Recruitment was conducted with assistance from ART nurses and case managers who reviewed patient records and identified individuals with diverse adherence histories (adherent, irregular, and previously lost to follow-up). Eligible patients were approached during clinic visits and invited to participate. Sampling continued until data saturation was achieved, defined as the point when no new themes emerged from interviews.

All ART nurses and case managers working in the two clinics during the study period were invited to participate in focus group discussions.

### Data collection

Data were collected between March and August 2024 using semi-structured interview and FGD guides. The guides were designed to elicit participants’ perspectives on factors influencing ART adherence, barriers to retention, and facilitators of continued engagement in care. Guides were refined after the first two interviews to improve clarity and flow; however, no substantive content changes were made.

- *Patient interviews*: Conducted face-to-face in Somali by trained qualitative researchers, lasting 45–75 min.
- *Provider FGDs*: Three FGDs with 12 healthcare workers (8 nurses, 4 case managers), lasting 90 min each.

All sessions were audio-recorded and supplemented by field notes. Participants chose interview times and locations.

Data were stored in locked cabinets at Banadir Hospital and password-protected servers at the Somali Ministry of Health. Data will be destroyed five years after publication.

### Data management and transcription

Interviews and FGDs were transcribed verbatim in Somali. Transcripts were reviewed for accuracy against recordings. Two bilingual translators translated transcripts into English, and discrepancies were resolved collaboratively. A third independent translator back-translated selected transcripts into Somali to ensure conceptual equivalence. A panel consisting of a qualitative researcher, a pharmacist, and a language expert reviewed the translations.

### Data analysis

Data analysis aimed to identify barriers and facilitators to ART adherence, including reasons for missed appointments and loss to follow-up. Data were analyzed thematically following Braun and Clarke’s six-step framework [15]. Transcripts were uploaded into *NVivo 12* [16] (QSR International, Melbourne, Australia).

Two independent coders (a social scientist and a physician) conducted open coding. Codes were grouped into categories and then abstracted into themes. Coding differences were resolved through discussion. Interviews and FGDs were analyzed separately and then compared for convergence. Representative quotes were selected to illustrate key findings.

### Ethics statement

The study was approved by the Somali Ministry of Health Research Ethics Committee. All interviews were conducted in private rooms to ensure confidentiality. FGDs were held in hospital auditoriums. Participants were informed of their right to withdraw at any time and were encouraged to stop or redirect discussion if they experienced distress. The study purpose was explained using standard information sheets, and written informed consent was obtained.

At the end of each session, participants who lacked transport fare received $5 USD equivalent to cover transport and time. Patients who disclosed non-adherence during the study were referred to case managers for counseling and support. Identifying information was not retained in transcripts.

## Results

### Study participants

A total of 22 patients and 12 healthcare providers (8 ART nurses and 4 case managers) participated in the study. Twenty-two face-to-face interviews and three focus group discussions were conducted in Banadir hospital, Mogadishu, Somalia.

### Characteristics of study participants

#### Patients

The mean age of the patients was 37 years (range: 22–52). More than half (55%) were women. 41% had no formal education, while 36% had completed primary school. Approximately half (46%) had previously defaulted and later re-engaged in ART care, while the remainder were consistently on follow-up. Among those on regular follow-up, 64% reported never missing appointments, while 36% admitted to occasional or frequent missed visits as showing Table 1.

**Table 1:** Socio-demographic and adherence characteristics of study participants (*n* = 34)

Characteristic	Patients (*n* = 22)	Providers (*n* = 12)
Mean age (years)	37 (22–52)	33 (27–42)
Female, n (%)	12 (55%)	8 (67%)
Education – none/primary/secondary	9/8/5	–
Ever defaulted from ART	10 (46%)	–
Follow-up consistency – regular/irregular	12 (55%)/10 (45%)	–
Missed appointments – never/occasionally or frequently	14 (64%)/8 (36%)	–
Years in ART service (mean)	–	5 years

#### Healthcare providers

The mean age of providers was 33 years. Two-thirds (67%) were women. Eight were ART nurses and four were case managers, with an average of five years of HIV clinic experience.

### Themes and categories

The themes that emerged were classified into two broad categories: barriers to ART adherence and facilitators of ART adherence. Each theme was further divided into categories, as presented below and summarized in Table 2.

**Table 2:** Emergent themes and categories influencing ART adherence and retention in care among HIV-positive adults in Somalia

Themes	Categories	Influence on Adherence
Patient-Related Factors	Stigma and discrimination (fear of disclosure, workplace stigma, social isolation)	Barrier
	Psychological factors (depression, denial, hopelessness)	Barrier
	Personal motivation and resilience (desire to live for children, education, work)	Facilitator
Socio-Cultural Factors	Religious and cultural beliefs – reliance on prayer/traditional healers	Barrier
	Religious coping – faith as motivation to survive and adhere	Facilitator
Economic Factors	Poverty and food insecurity	Barrier
	Transport costs/lack of fare	Barrier
Health System Factors	Stock-outs of ART drugs	Barrier
	Long waiting times at clinics	Barrier
	Lack of confidentiality and privacy	Barrier
	Positive patient–provider relationship (trust, counseling, respect)	Facilitator
Social Support Factors	Family support (reminders, financial help, emotional encouragement)	Facilitator
	Peer support networks (PLHIV groups, shared experiences)	Facilitator
	NGO support (food rations, transport assistance)	Facilitator

### Theme I: barriers to ART adherence

#### Category I: stigma and discrimination

Stigma was the most frequently cited barrier to consistent ART adherence. Participants described both community and interpersonal stigma that led to secrecy, medication hiding, and missed doses. Many adults living with HIV feared being recognized at local clinics or seen taking their medication. This fear often led them to seek care in distant facilities or to interrupt treatment when concealment was difficult.

One patient shared:

“*When I travel to the clinic near my house*,* I feel everyone is watching me. That fear makes me go to another district*,* but sometimes I don’t have money to go far*.” (Female, 26 years, patient)

Another participant explained how social settings contributed to non-adherence:

“*I miss doses when I am with friends. They don’t know about my illness*,* and I’m afraid they will start gossiping if they see me with pills.*” (Male, 30 years, patient)

Health providers also reported that patients took extra precautions to hide their medication at home:

“*Some patients hide their bottles under mattresses or in walls. They fear family members might see them*.” (Female, nurse, FGD1)

The impact of stigma extended beyond the home and community into workplaces, where fear of discrimination or job loss influenced adherence behaviour. Some patients delayed or skipped doses to avoid taking medication during working hours.

“*In my job, if the manager finds out I have HIV, I am sure they will remove me. That fear makes me delay my pills*.” (Male, 33 years, patient)

Others described witnessing severe consequences of disclosure, which reinforced secrecy and disrupted adherence.

“*A young woman I counselled lost her domestic work because her employer saw her medicine. Since then, she hides, and sometimes she skips doses*.” (Male, case manager, FGD2)

Across these accounts, stigma operated at multiple levels i.e. community, household, and workplace producing constant anxiety about disclosure. These fears discouraged open treatment-taking, interfered with medication routines, and undermined regular clinic attendance, highlighting how social discrimination continues to constrain adherence among adults living with HIV.

#### Category II: religious and cultural beliefs

Religion and culture exerted a profound but ambivalent influence on ART adherence. Many participants interpreted illness and healing through spiritual frameworks that either undermined or reinforced adherence behaviour. Some adults living with HIV described substituting their medication with religious rituals or traditional healing, believing divine intervention alone could bring recovery. These beliefs often led to temporary discontinuation of ART and subsequent clinical deterioration.

A participants reported:

“*The sheikh gave me special Qur’anic water and told me Allah would heal me. I stopped my tablets for two months until I became sick again*.” (Male, 34 years, patient)

“*I went to a herbal healer who said HIV is a punishment and medicine cannot help. I wasted many months before coming back*.” (Female, 29 years, patient)

Health-care workers also observed that some patients returned to clinics in poor health after following the advice of spiritual healers, reflecting the continuing power of religious authority in shaping health decisions.

“*We see patients returning very sick after stopping medicine because of religious leaders’ advice*.” (Female, nurse, FGD2)

However, religion also served as a vital source of strength and hope for others. Participants who viewed faith and medicine as complementary described prayer as a daily coping mechanism that encouraged discipline in treatment-taking. For these individuals, spirituality became a facilitator rather than a barrier to adherence.

*“I pray before every dose. It gives me hope that God wants me to live*,* so I never miss my pills.”* (Female, 40 years, patient)

“*During Ramadan I take my medicine with the evening meal. My faith makes me strong to continue*.” (Male, 38 years, patient)

Across these accounts, religion functioned as a double-edged sword—both impeding and enabling adherence depending on personal interpretation and community influence. The contrasting experiences highlight how spiritual beliefs operate at the community level to shape patients’ understanding of illness, moral responsibility, and treatment commitment. Integrating faith leaders into HIV education and counseling may therefore transform religious conviction from a source of risk into a driver of adherence.

#### Category III: economic hardship

Economic hardship emerged as a persistent structural barrier to consistent ART adherence. Participants described how poverty, unemployment, and food insecurity directly disrupted their treatment routines. The inability to afford transport to clinics or to eat before taking medication often forced patients to skip doses or delay appointments. These material constraints interacted with social and system-level factors such as stigma and long waiting times, compounding the challenges of staying on treatment.

“*Sometimes I don’t eat all day. Taking pills without food makes me feel sick, so I skip them*.” (Female, 41 years, patient)

“*If I have no bus fare, I miss my appointment. I can’t walk that long distance*.” (Male, 36 years, patient)

Health-care providers confirmed that loss of food or transport assistance frequently led to disengagement from care, with some patients returning only when external aid resumed.

“*Some patients disappear when NGO food support stops. They come back only if they hear food has returned*.” (Female, case manager, FGD3)

Food insecurity also generated misinformation about the dietary requirements for taking ART, creating another layer of risk for poor adherence.

“*People believe you must eat meat or eggs with medicine. If they cannot afford it, they stop the pills*.” (Male, nurse, FGD1)

Together, these accounts illustrate how economic deprivation operates as both a practical and psychological obstacle to treatment. Poverty not only limits access to clinics and nutrition but also undermines confidence in the feasibility of long-term therapy. Sustained adherence in such contexts therefore depends on addressing patients’ basic livelihood and nutritional needs alongside medical care.

#### Category IV: health system challenges

Barriers within the health system further undermined adherence and retention in care. Both patients and providers emphasized that frequent drug stock-outs, overcrowded clinics, and long waiting times disrupted medication schedules and discouraged attendance. For many, these issues translated into treatment interruptions despite a strong desire to remain adherent.

“*One time I came, and they told me medicine was finished. I missed for two weeks*.” (Female, 32 years, patient)

“*We come at 6 a.m. and wait until afternoon. Some people just leave without collecting drugs*.” (Male, 28 years, patient)

Lack of privacy at ART clinics also contributed to avoidance and fear of disclosure. Patients reported that visible clinic entry points made them vulnerable to community judgment, deterring some from seeking care.

“*Everyone can see when you enter the ART room. That makes people ashamed and some refuse to come*.” (Female, 25 years, patient)

Health-care providers echoed these concerns, noting that limited space, staff shortages, and high patient load often compromised the quality of counseling and individualized care.

“*The clinic is overcrowded. We try our best, but sometimes we write prescriptions quickly without proper discussion*.” (Male, case manager, FGD2)

Collectively, these accounts reveal how weaknesses in service delivery and infrastructure interact with social stigma to erode patient confidence. Addressing these systemic issues—ensuring consistent drug supply, reducing waiting times, and protecting privacy—is essential to sustain adherence and foster trust between patients and providers.

#### Category V: psychological factors

At the individual level, psychological distress emerged as a major barrier to consistent ART adherence. Feelings of depression, hopelessness, and denial of diagnosis were common, particularly in the early stages of treatment. Participants described emotional exhaustion and loss of motivation that led them to stop taking medication or miss follow-up appointments.

“*Sometimes I feel there is no future. I stop my medicine because I think I will die anyway*.” (Male, 29 years, patient)

“*At first, I refused to believe I had HIV. For almost one year, I didn’t take the medicine properly*.” (Female, 27 years, patient)

Health providers corroborated these experiences, observing that psychological distress often preceded treatment discontinuation.

“*Many who are lost to follow-up are struggling with sadness or mental stress. They don’t see the point of continuing*.” (Female, nurse, FGD3)

These narratives highlight the emotional burden carried by adults living with HIV and how mental health challenges can quietly undermine otherwise motivated patients. Integrating psychosocial counseling and mental health support into routine HIV care could help mitigate these barriers and promote long-term adherence.

### Theme II: facilitators of ART adherence

#### Category I: family and social support

Participants consistently reported that family support was one of the strongest facilitators of ART adherence. Spouses, children, and close relatives reminded them to take medication, prepared meals, and provided emotional encouragement. These acts of care created accountability and routine that strengthened treatment commitment.

“*My wife always reminds me and prepares food before I take the pills*.” (Male, 42 years, patient)

“*My daughter brings me water and medicine every evening. Without her, I would forget*.” (Female, 45 years, patient)

Participants and providers also shared that assistance from nongovernmental organizations—such as food packages or hygiene items—reinforced adherence by easing household stress.

“*NGOs help with food and soap. Patients who get this support rarely miss appointments*.” (Male, case manager, FGD1)

Across these narratives, familial and community assistance functioned as practical and emotional scaffolding. Regular reminders, shared meals, and tangible aid transformed ART from an individual responsibility into a collective family effort.

#### Category II: religious coping

Participants shared that while some religious teachings had previously undermined adherence, many drew strength from faith to persevere. They viewed medicine as part of divine provision and prayer as a sustaining ritual that offered hope and discipline.

“*I believe Allah gave knowledge to doctors to make medicine. That’s why I take my pills and pray together*.” (Female, 36 years, patient)

“*Faith gives patients courage. They say, ‘Allah kept me alive, so I will continue*.’” (Female, nurse, FGD2)

Religion therefore acted not merely as belief but as a coping mechanism that helped patients maintain psychological balance. Integrating faith-affirming messages into counseling could reinforce this positive alignment between spirituality and adherence.

#### Category III: positive patient–provider relationships

articipants reported that trust and respectful communication with health-care workers encouraged consistent attendance and adherence. Feeling listened to and treated with dignity strengthened motivation and reduced anxiety about follow-up visits.

“*The nurse listens to me and encourages me. Because of her, I continue*.” (Male, 32 years, patient)

“*When providers respect us, patients feel valued. They come back*.” (Male, case manager, FGD1)

Such statements illustrate how relational quality within clinics can transform the patient experience from obligation to partnership, promoting sustained engagement in care.

#### Category IV: peer support networks

Participants shared that peer support groups reduced isolation and replaced secrecy with solidarity. Meeting others living healthily with HIV fostered belonging, normalized treatment, and provided informal accountability.

“*In our support group, we share experiences. Seeing others living healthy gives me strength*.” (Male, 37 years, patient)

“*Patients who attend group meetings are more committed to pills*.” (Female, nurse, FGD3)

These accounts highlight the social value of peer networks as living testimony that adherence enables normal, productive lives, reinforcing optimism and commitment among members.

#### Category V: personal motivation and resilience

Participants frequently reported strong personal motivations for adherence, often grounded in family responsibility, hope for the future, and the will to survive. Many described taking medication as an act of love and duty toward their children, viewing continued treatment as the only way to safeguard their families’ well-being.

“*I take my medicine for my children. If I die, they have no one*.” (Male, 38 years, patient)

Others linked adherence to specific goals or life aspirations, emphasizing how family bonds provided meaning and discipline in their treatment routines.

“*I want to see my son finish school. That is why I never stop*.” (Female, 35 years, patient)

Several participants also described resilience that extended beyond family motives—a personal determination to live despite fatigue or hardship. Their narratives portrayed ART adherence as both a physical and psychological commitment to life itself.

“*Even when I feel tired, I push myself because I know my life depends on these pills*.” (Male, 44 years, patient)

Across these accounts, adherence was seen not merely as a medical obligation but as a moral and emotional endeavor sustained by hope, responsibility, and self-belief. Personal motivation and resilience thus acted as powerful protective factors, enabling patients to overcome the daily struggles associated with poverty, stigma, and ill health.

## Discussion

This qualitative study explored multilevel barriers and facilitators to ART adherence among HIV-positive adults in Somalia. Using the socio-ecological lens, the findings revealed that adherence is shaped by a complex interaction of factors operating at individual, interpersonal, community, and health-system levels. The major barriers identified included stigma and discrimination, religious and cultural beliefs (i.e., reliance on prayer or traditional healers), economic hardship (food insecurity and transport difficulties), health-system challenges (stock-outs, long waits, lack of confidentiality), and psychological factors (depression and denial). Facilitators included strong family and social support, religious coping, trusting patient–provider relationships, peer support networks, and personal motivation and resilience, particularly among patients driven by family responsibility and hope for their children’s future.

At the individual level, psychological distress and hopelessness were central obstacles to consistent adherence. Depression and denial frequently led to missed doses or disengagement from care. These experiences resonate with prior work in Sub-Saharan Africa showing that poor mental health impedes adherence and that integrated psychosocial interventions can improve outcomes [10]. Group interpersonal therapy in Senegal, for instance, successfully reduced depressive symptoms among PLHIV and improved adherence, emphasizing the need for culturally adapted mental health components within HIV programs.

At the interpersonal level, family, peers, and providers played protective roles. Family reminders, emotional encouragement, and financial help sustained treatment routines, while peer networks offered social belonging and motivation. Similar findings were reported in Uganda, where social isolation significantly predicted non-adherence, and same-gender peer ties improved retention [13]. Our findings reaffirm that interpersonal relationships serve as critical adherence anchors, particularly in settings of stigma and poverty.

At the community level, stigma and religion emerged as intertwined influences. Stigma was pervasive across households, workplaces, and social spaces, producing secrecy and avoidance of clinics. These findings echo reports from Ethiopia and across East Africa, where stigma, poverty, and weak health systems jointly constrain adherence [7]. In other African contexts, such as Tanzania and Uganda, discrepancies between patient and provider perceptions—patients stressing economic hardship while providers emphasized stigma—underscore the need to align clinical interventions with lived experiences [17]. Moreover, religious beliefs had dual effects: while some patients discontinued ART in favor of prayer or traditional healing, others used faith as a coping resource. This pattern parallels evidence from South Africa showing that training traditional healers to provide HIV counseling can reduce stigma and improve linkage to care [18]. Engaging Somali religious and community leaders could similarly transform cultural beliefs from barriers into facilitators.

At the structural and health-system level, weaknesses such as drug stock-outs, long waiting times, and poor confidentiality eroded trust in services and disrupted adherence. Economic hardship and food insecurity compounded these challenges, as participants struggled to take medication without meals or afford transport. Comparable findings in other low-resource settings highlight how poverty intersects with fragile health infrastructure to hinder sustained treatment access [7, 17]. A recent cross-sectional study in Mogadishu also found that living alone, perceived stigma, and co-morbid conditions predicted non-adherence, reinforcing the relevance of these structural and psychosocial determinants [2].

Collectively, these findings align with regional evidence that adherence is not a purely individual behavior but a social and structural phenomenon influenced by intersecting layers of constraint and support [10, 13, 17]. Strengthening adherence in Somalia therefore requires a holistic, culturally sensitive response that engages all ecological levels.

Reducing stigma through community awareness and inclusion of religious leaders can reshape social attitudes toward HIV. Integrating mental health screening and counseling into ART clinics could mitigate depression and denial, while ensuring consistent ART supply, shortening waiting times, and enhancing privacy would rebuild confidence in the health system. Expanding social protection measures—such as food rations and transport subsidies—would address the economic precarity that directly affects adherence. Finally, fostering peer-led and family-centered adherence programs may capitalize on existing community solidarity and religious resilience to sustain long-term engagement in care.

## Limitations

As a qualitative study, these findings provide depth of understanding rather than statistical generalizability. Participants were all enrolled in ART programs and regularly engaged in care, which may have excluded the perspectives of individuals who are completely disengaged from treatment or who have never initiated ART. Moreover, the study relied on self-reported experiences that could be influenced by social desirability or recall bias. Future research should include broader populations—such as those lost to follow-up—and test context-specific interventions that address the barriers identified in this study.

## Conclusion

This study highlights how multilevel social, cultural, economic, and health-system factors collectively shape ART adherence among adults living with HIV in Somalia. Stigma, poverty, and structural weaknesses within the healthcare system continue to undermine adherence, while family support, personal motivation, and positive relationships with healthcare providers offer strong facilitating forces.

Rather than single-focus solutions, these findings point to the need for integrated, culturally sensitive, and community-based strategies that strengthen both psychosocial and structural support for patients. Interventions that combine stigma reduction, economic and nutritional assistance, mental health counseling, and faith-based engagement may help improve adherence and long-term treatment outcomes. Ultimately, addressing these interconnected barriers through multi-sectoral collaboration will be essential to sustain Somalia’s HIV response and enhance the quality of life for people living with HIV.

## Data Availability

All data generated or analyzed during this study are included in this published article and its supplementary information files.
